# Supplementary material for: Global disparities in SARS-CoV-2 genomic surveillance
Source: Nat Commun. 2022 Nov 16;13:7003. doi: 10.1038/s41467-022-33713-y (PMC9667854; doi:10.1038/s41467-022-33713-y)
Supplement: Supplementary file 1 — Supplementary Information [file 41467_2022_33713_MOESM1_ESM.pdf]

## Supplementary Materials

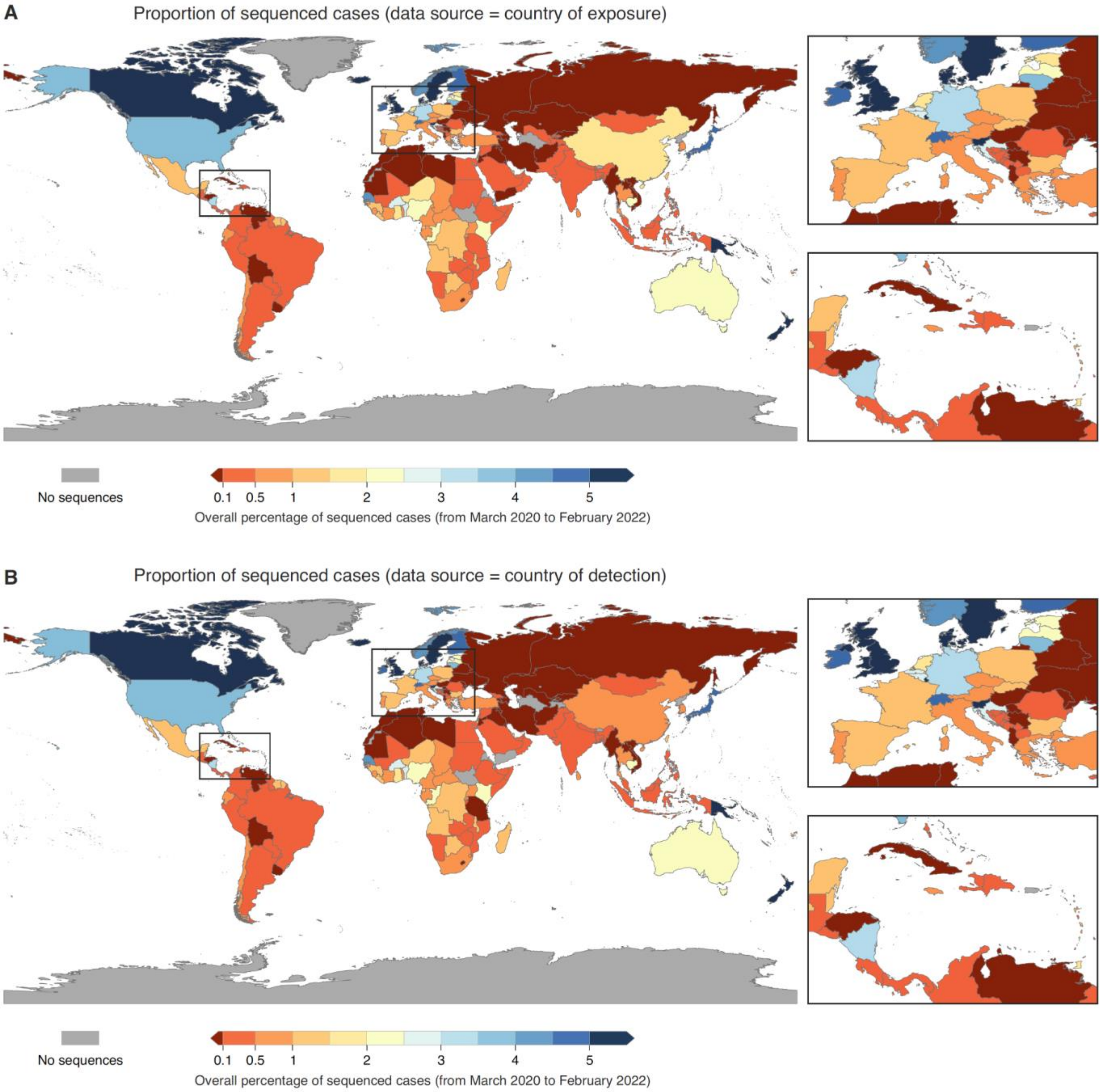


**Fig. S1.** Overall percentage of sequenced cases per country, between March 2020 and February 2022, based on metadata submitted to GISAID up to March 18th, 2022. The data shown here are the same used in Figure 1 to display weekly sequencing percentages. (A) Sequencing percentages observed when "country of exposure" is used as data source for defining the geographic origin of genomes, to reflect the locations where infections started (instead of where cases were detected). (B) Sequencing percentages observed when "country of sampling" is used as data source for defining the geographic origin of genomes, to reflect the locations where the infections were detected and where the cases were sequenced.

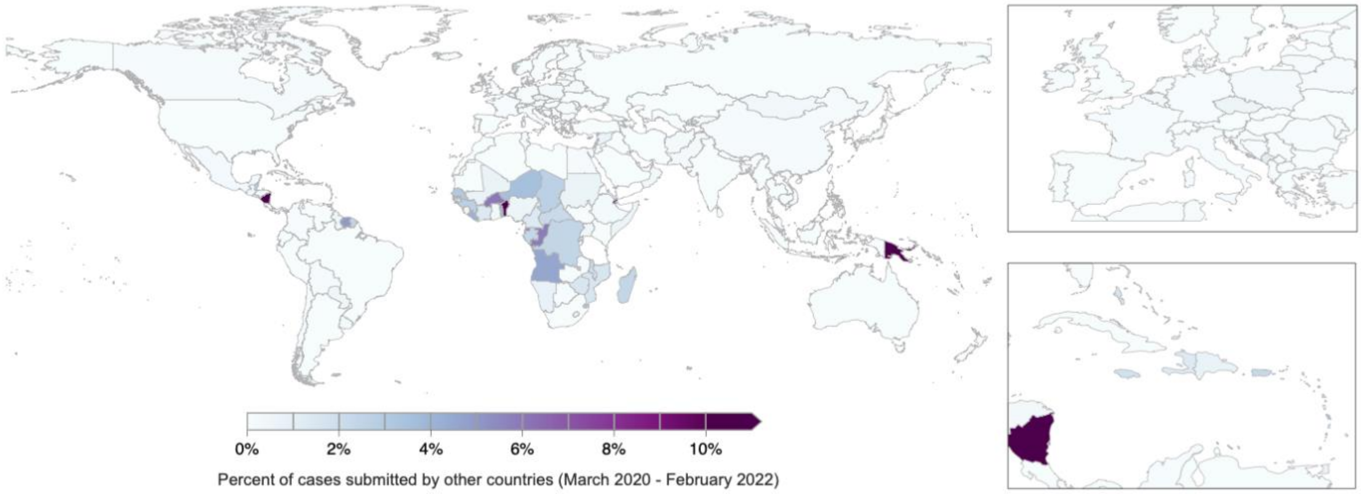


**Fig. S2.** Countries that rely mostly on other countries' capacity for genome sequencing and submission. Countries that rely on external resources are highlighted with shades of purple, based on the percentage of their cases that were sequenced and submitted by other countries.

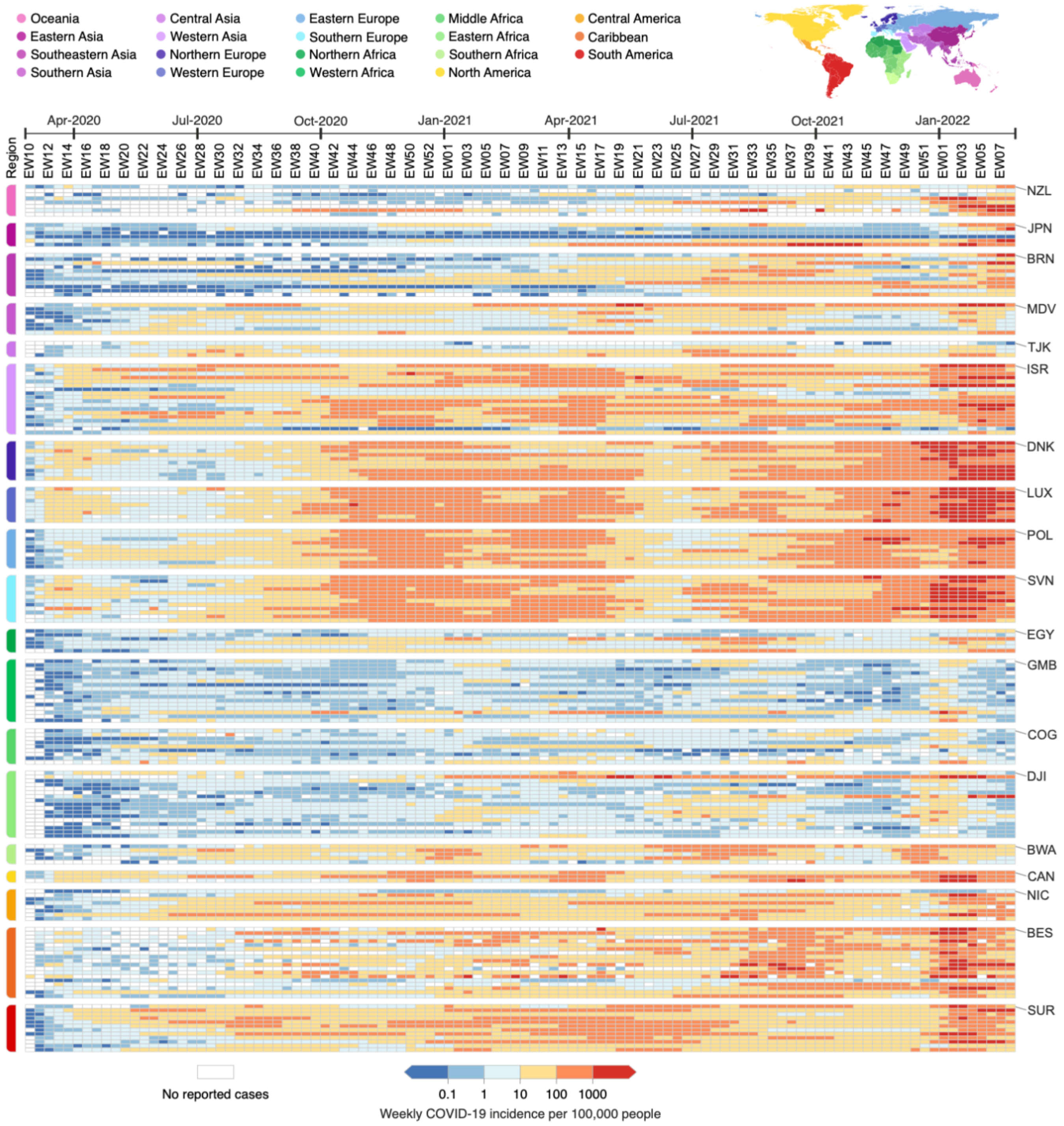


**Fig. S3.** Weekly COVID-19 incidence (cases per 100,000 people), using the same data displayed in Figure 2B. In the manuscript, we refer to incidence levels following the US CDC metric, in place up to late 2021: low incidence (<10 weekly cases per 100.000 people, blue shades); moderate/substantial (10-99, yellow); high (>100 weekly cases per 100.000 people, orange and red shades). Countries are grouped in regions according to the UNSD geoscheme, and countries are ordered as in Figure 1, and those with the highest overall proportion of sequenced cases in each region are highlighted using the ISO 3166-1 nomenclature: NZL = New Zealand; JPN = Japan; BRN = Brunei; MDV = Maldives; TJK = Tajikistan; ISR = Israel; DNK = Denmark; LUX = Luxembourg; POL = Poland; SVN = Slovenia; EGY = Egypt; GMB = Gambia; COG = Republic of the Congo; DJI = Djibuti; BWA = Botswana; CAN = Canada; NIC = Nicaragua; BES = Bonaire; and SUR = Suriname.

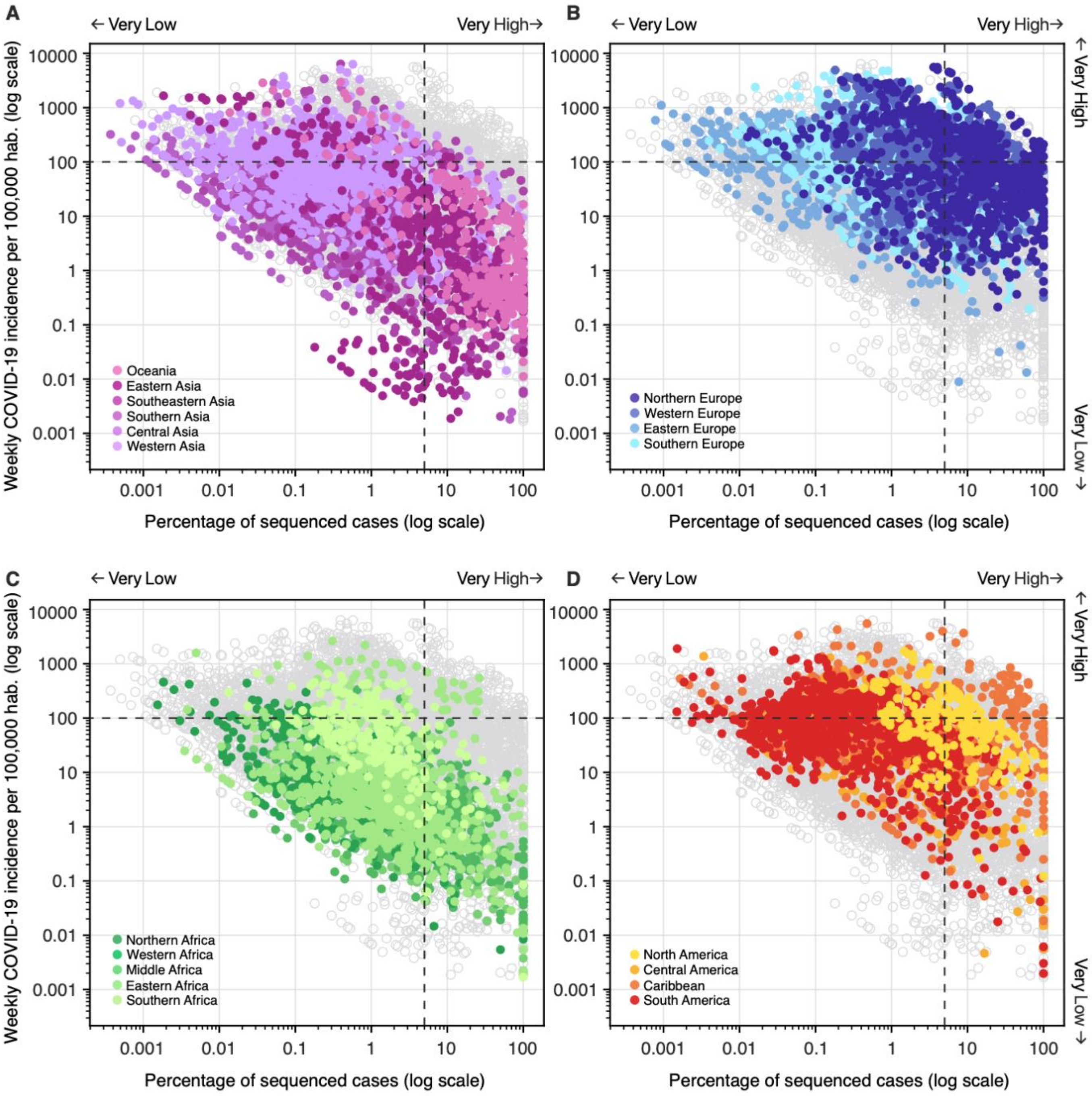


**Fig. S4.** Correlation between weekly COVID-19 incidence per 100,000 people, and percentage of sequenced cases in (A) Oceania & Asia, (B) Europe, (C) Africa and (D) the Americas, using the same data displayed in Figure 2B, where each point represents an epidemiological week in a country. Vertical dashed lines represent the threshold of 5% sequenced cases, while the horizontal line marks 100 cases per 100,000 people (high COVID-19 incidence).

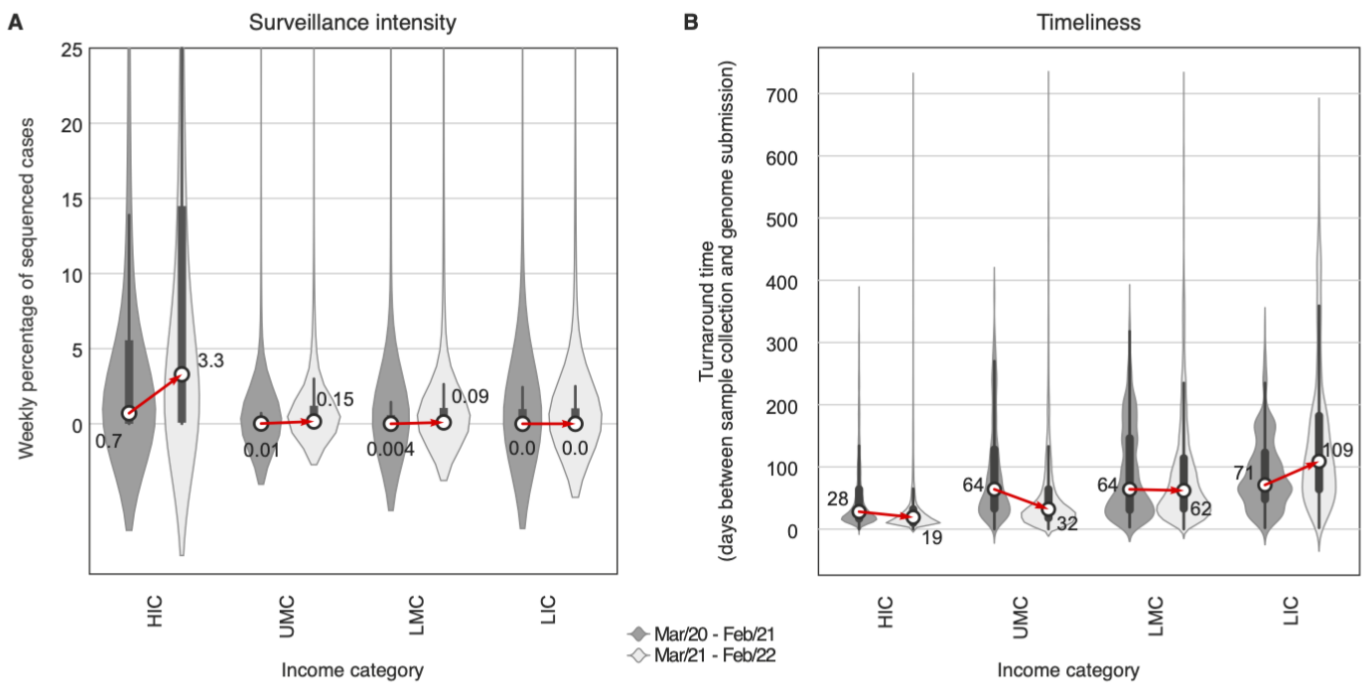


**Fig. S5.** Changes in sequencing intensity and timeliness between the first and second years of the COVID-19 pandemic. (A) Changes in the percentage of weekly sequenced cases, and (B) turnaround time in countries from different income categories: HIC - high income class, UMC - upper middle income class, LMC - low middle income class, LIC - lower income class. The data displayed here are the same shown in Fig. S4 and S6, respectively (n = 8,947,455 genomes). Genomes are grouped by year of submission, and the corresponding median values are highlighted. The elements in the violin plots represent the median (white circles), the interquartile range (black rectangles) and the minimum and maximum data points in the data sets (black vertical lines). The red arrows highlight the changes in the median surveillance intensity and timeliness between the first and second year of pandemic.

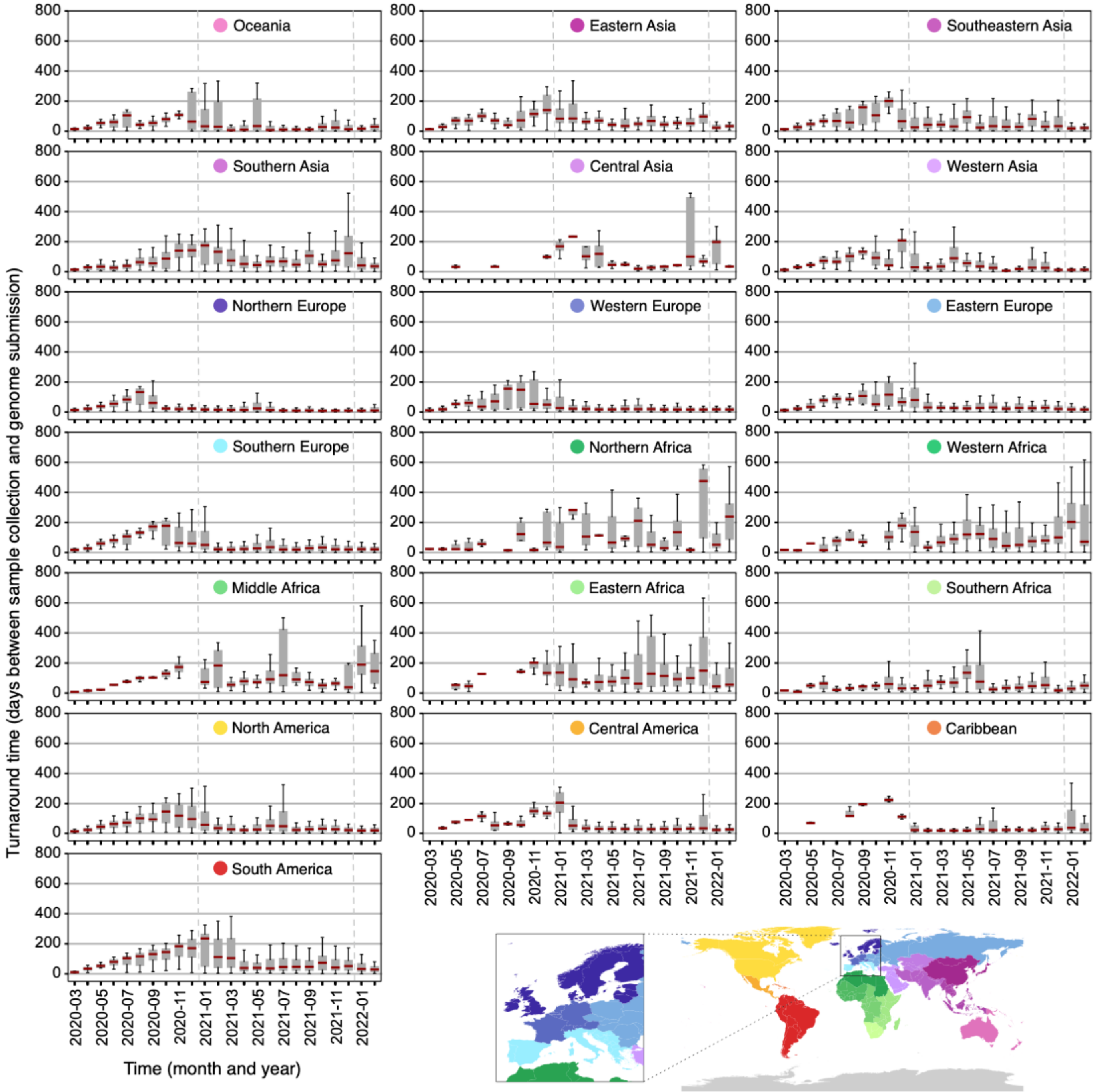


**Fig. S6.** Turnaround time across geographic regions. Delays between sample collection and genome submission (genomes grouped by their month of submission) in different geographic regions, for genomes collected from epidemiological week (EW) 10 of 2020 (March 1st, 2020) to EW 8 of 2022 (February 26th, 2022), based on metadata submitted to GISAID up to March 18th, 2022 (n = 8,947,455 genomes). Red markers indicate the median TAT in each month. The elements in the barplots represent the median (red marks), the interquartile range (gray rectangles) and the minimum and maximum data points in the data sets (black vertical lines).

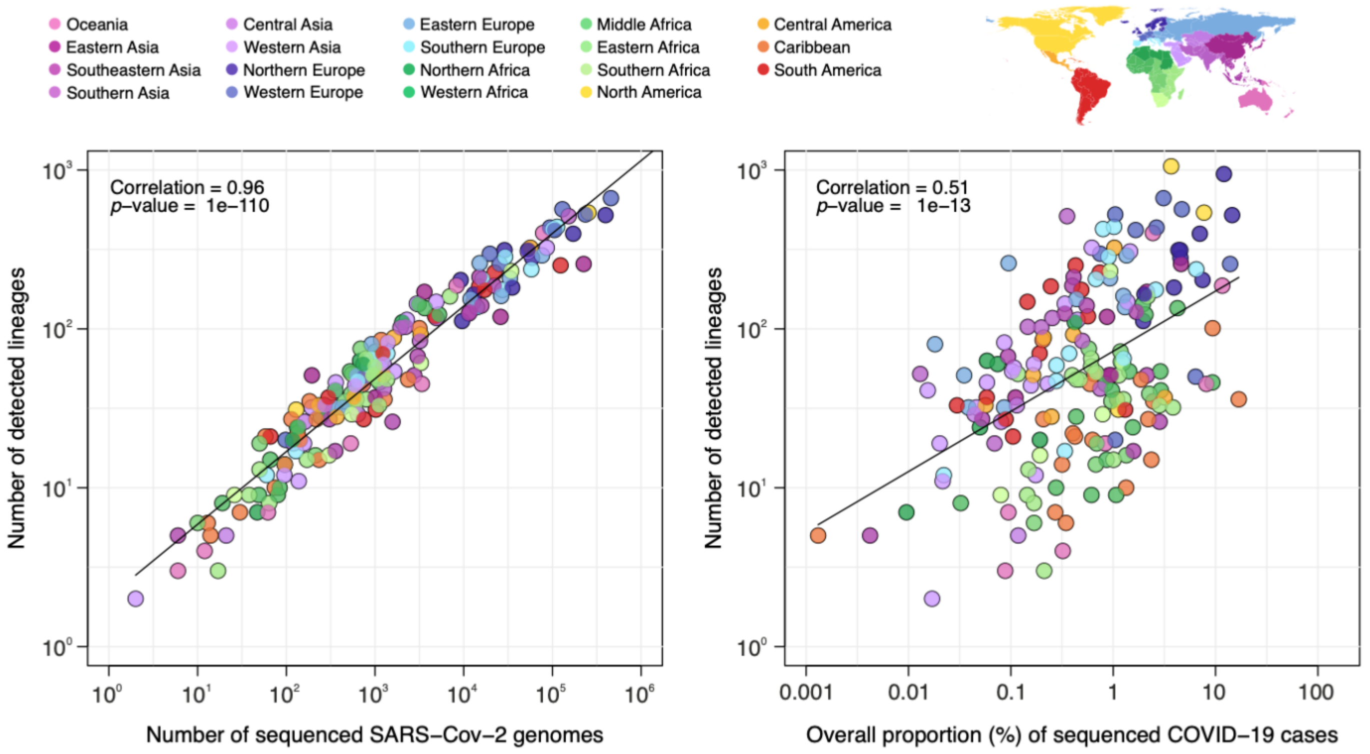


**Fig. S7.** Correlation between $\log_{10}$-transformed number of detected lineages and $\log_{10}$-transformed **(A)** number of sequenced genomes (slope = 0.45, CI = (0.44, 0.47), t-value = 50.27) and **(B)** percentages of sequenced cases per country (slope = 0.37, CI = (0.28, 0.47), t-value = 8.046).

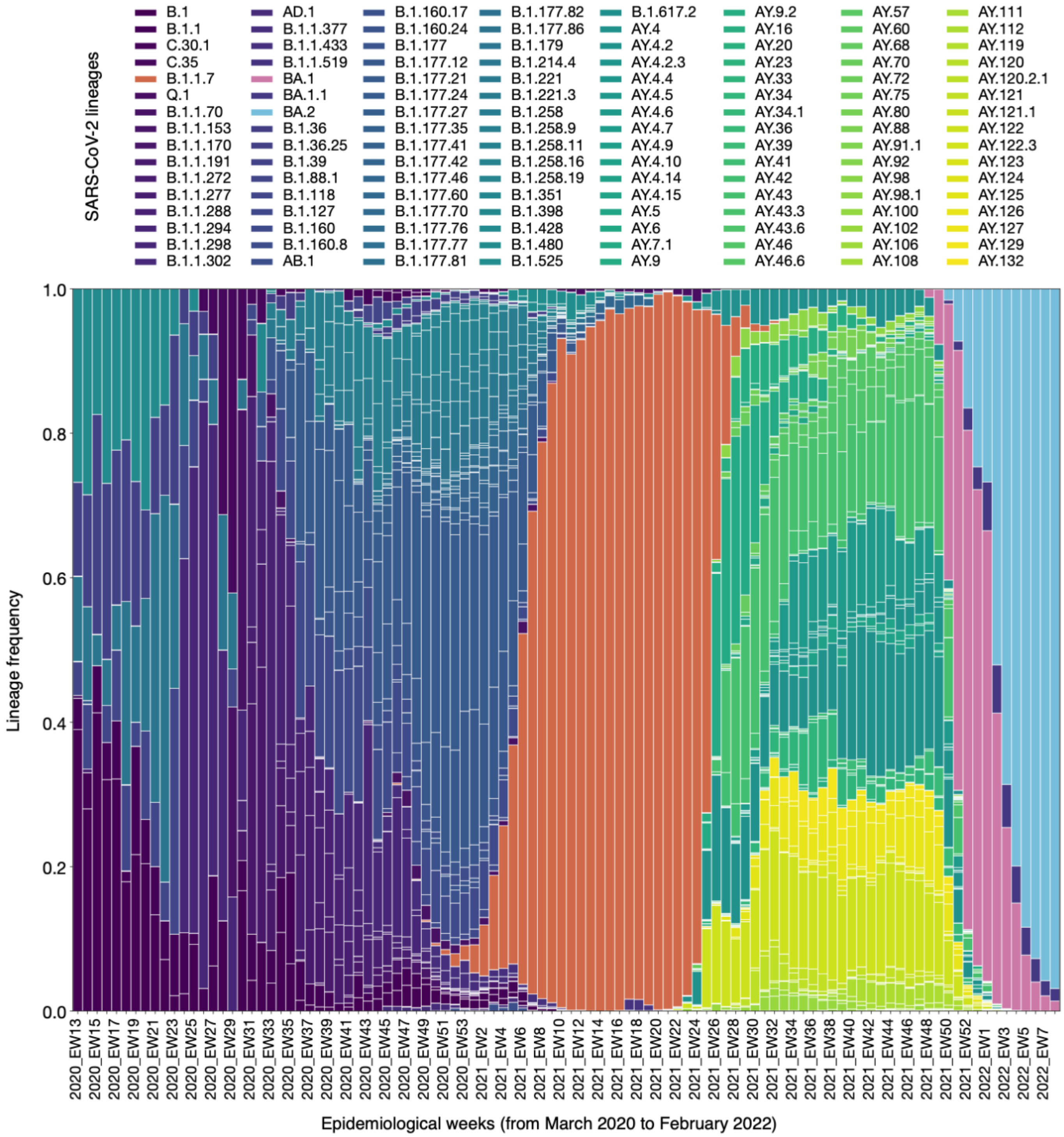


**Fig. S8.** Relative frequency of SARS-CoV-2 lineages detected in Denmark between March 2020 and February 2022 (grouped by epiweeks, based on collection dates). In this period the country sequenced more than 14% of its reported cases, on average, and this dataset was used as the 'ground truth' for the simulations of probabilities of lineage detection shown in Figure 3B-G.

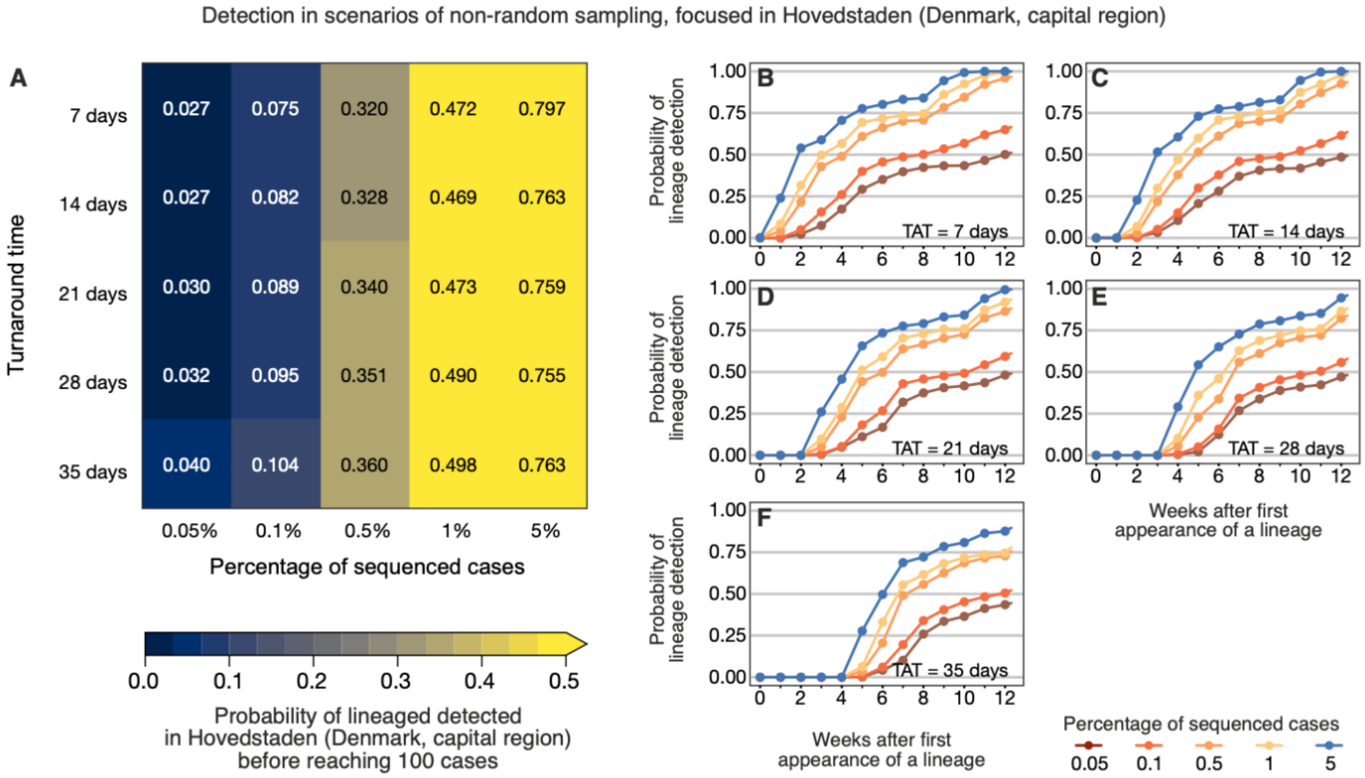


**Fig. S9.** Detection of SARS-CoV-2 lineages under different genomic surveillance scenarios, under non-random sampling focused in the most populous region of a country (in this example, using data from Hovedstaden, capital region of Denmark). (A) Relative importance of decreasing genome sequencing turnaround time (TAT) versus increasing sequencing percentage, measured as probability that a lineage found in Hovedstaden (in simulated datasets) was detected before it had reached 100 cases in all of Denmark (as in **Fig. 3**). (B-F) Probability of detecting any of the top 10 most prevalent lineages of Denmark in Hovedstaden, considering TATs of 7, 14, 21, 28 and 35 days.

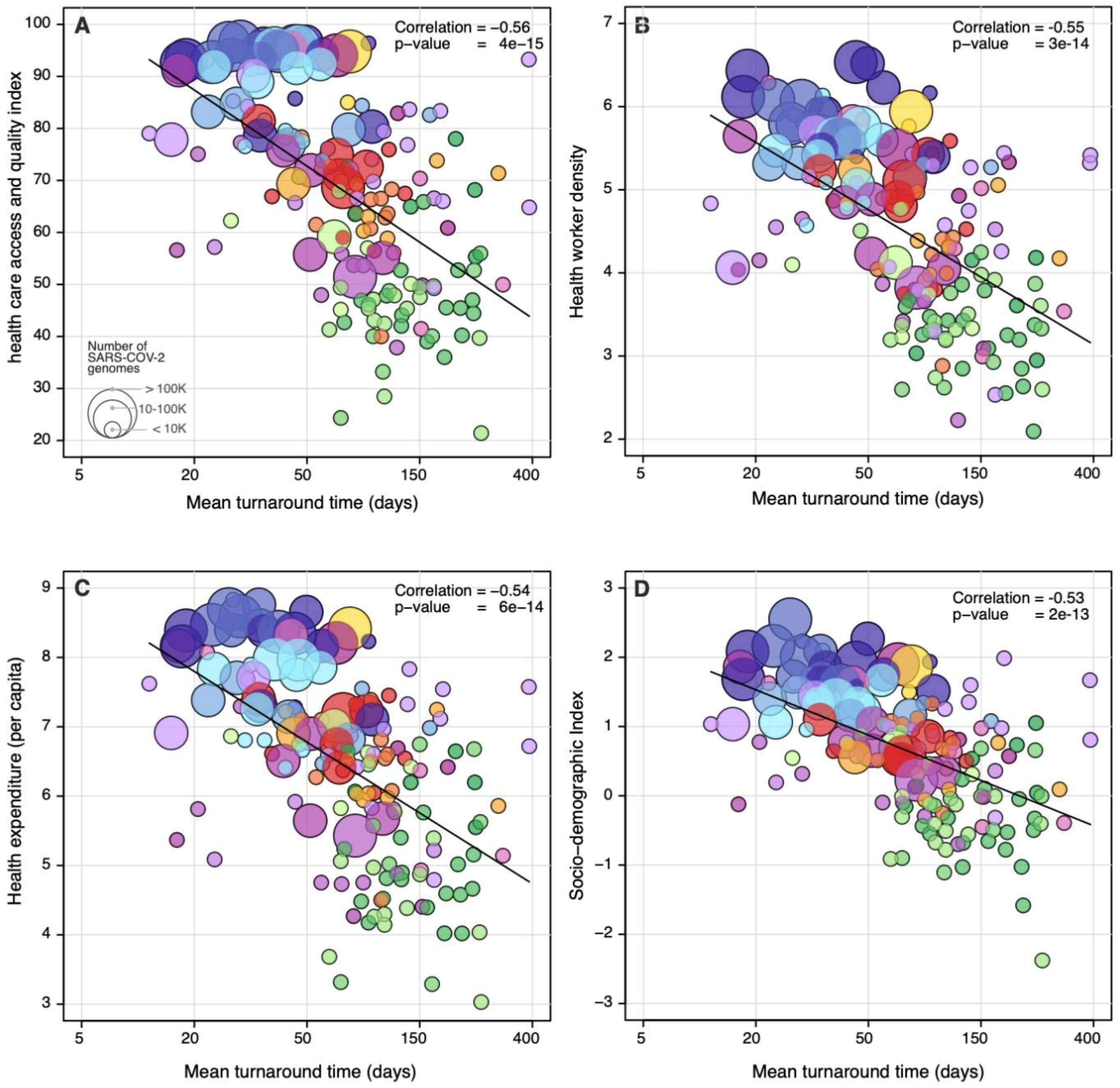


**Fig. S10.** Covariates that show the highest negative correlation with the mean turnaround time. (A) Health care access and quality (slope = -18.046, CI = (-22.13, -13.95), t-value = -8.64); (B) Health worker density (slope = -0.81059, CI = (-1.00, -0.61), t-value = -8.32); (C) Health expenditure (per capita) (slope = -1.0205, CI = (-1.26, -0.77), t-value = -8.22); (D) Socio-demographic Index (slope = -0.65, CI = (-0.81, -0.49), t-value = -8.05). The colour scheme of geographic regions is the same used in Figure 1. The solid lines show the linear fit.
