# Supplementary material for: Global disparities in SARS-CoV-2 genomic surveillance
Source: Nat Commun. 2022 Nov 16;13:7003. doi: 10.1038/s41467-022-33713-y (PMC9667854; doi:10.1038/s41467-022-33713-y)
Supplement: Supplementary file 3 — Description of Additional Supplementary Files [file 41467_2022_33713_MOESM3_ESM.pdf]

Description of Additional Supplementary Files

File Name: Supplementary Data 1
Description: Percentage of sequenced COVID-19 cases per country per epidemiological week (EW), between March 2020 and February 2022 (based on metadata submitted to GISAID up to March 18th, 2022). The data shown here are the same used in Figure 1 to display weekly sequencing percentages. X = No cases; Code = ISO 3166-1 alpha-3; Income class according to the World Bank classification: low income class (LIC); lower middle income class (LMC); upper middle income class (UMC); and high income class (HIC). Frequency of sampling = Proportion of weeks with at least one genome.

File Name: Supplementary Data 2
Description: List of countries that mostly relied on other countries to have their COVID-19 cases sequenced.

File Name: Supplementary Data 3
Description: Sequencing statistics for countries in the study, with data submitted up to March 18th, 2022, corresponding to the collectionn period from March 2020 to February 2022. Code = ISO 3166-1 alpha-3. Income category according to the World Bank classification: low income countries (LIC); lower middle income countries (LMC); upper middle income countries (UMC); and high income countries (HIC).

File Name: Supplementary Data 4
Description: Key surveillance characteristics with a split by income class. We provide summary statistics of observed surveillance characteristics for each group of countries, defined by their income class. HIC: high income class, UMC: upper middle income class, LMC: low middle income class, LIC: lower income class, non-HIC: combined UMC, LMC and LIC.

File Name: Supplementary Data 5
Description: Typical country profiles characterized by covariates. We provide typical values of covariates which characterize capacity and coordination abilities for each group of countries, linked to their income level. HIC: high income class, UMC: upper middle income class, LMC: low middle income class, LIC: low income class. Abbreviations in the Table: Gross domestic product - GDP, Financing Global Health - FGH, lag-distributed income - LDI, total fertility rate - TFR, The Global Burden of Disease Study - GBD, Sustainable Development Goals - SDGs, International Standard Classification of Occupations - ISCO, Out-of-pocket - OOP, Purchasing power parity (dollars) - PPP.

File Name: Supplementary Data 6
Description: Correlations of country-level covariates with the percentage of sequenced COVID-19 cases for the entire duration of the study period (March 2020 - February 2022). 'Transformation' column denotes the transformation applied to the corresponding covariate before assessing the correlation; the p-value column shows significance of the slope in a linear model. The correlation is Pearson's correlation; p-values are reported based on the t-statistic using two-sided hypothesis, with the null hypothesis being that the slope of the linear trend is zero. There was no need for multiple comparison adjustments. Abbreviations

in the Table: Gross domestic product - GDP, Financing Global Health - FGH, lag-distributed income - LDI, total fertility rate - TFR, The Global Burden of Disease Study - GBD, Sustainable Development Goals - SDGs, International Standard Classification of Occupations - ISCO, Out-of-pocket - OOP, Purchasing power parity (dollars) - PPP.

File Name: Supplementary Data 7
Description: Correlations of country-level covariates with the mean turnaround time based on data from the entire duration of the study period (March 2020 - February 2022). 'Transformation' column denotes the transformation applied to the corresponding covariate before assessing the correlation (Pearson's correlation); the p-value column shows significance of the slope in a linear model, reported based on the t-statistic using two-sided hypothesis, with the null hypothesis being that the slope of the linear trend is zero. There was no need for multiple comparison adjustments.

File Name: Supplementary Data 8
Description: GISAID Supplemental Table is accessible at https://doi.org/10.55876/gis8.220330me.
